# Interaction between moxifloxacin and Mcl-1 and MITF proteins: the effect on growth inhibition and apoptosis in MDA-MB-231 human triple-negative breast cancer cells

**DOI:** 10.1007/s43440-022-00407-7

**Published:** 2022-09-01

**Authors:** Artur Beberok, Jakub Rok, Zuzanna Rzepka, Krzysztof Marciniec, Stanisław Boryczka, Dorota Wrześniok

**Affiliations:** 1 Department of Pharmaceutical Chemistry, Faculty of Pharmaceutical Sciences in Sosnowiec, Medical University of Silesia, Jagiellońska 4, 41-200 Sosnowiec, Poland; 2 Department of Organic Chemistry, Faculty of Pharmaceutical Sciences in Sosnowiec, Medical University of Silesia, Jagiellońska 4, 41-200 Sosnowiec, Poland

**Keywords:** Breast cancer, Moxifloxacin, In silico analysis, Cellular homeostasis

## Abstract

**Background:**

Microphthalmia-associated transcription factor (MITF) activates the expression of genes involved in cellular proliferation, DNA replication, and repair, whereas Mcl-1 is a member of the Bcl-2 family of proteins that promotes cell survival by preventing apoptosis. The objective of the present study was to verify whether the interaction between moxifloxacin (MFLX), one of the fluoroquinolones, and MITF/Mcl-1 protein, could affect the viability, proliferation, and apoptosis in human breast cancer using both in silico and in vitro models.

**Methods:**

Molecular docking analysis (in silico), fluorescence image cytometry**,** and Western blot (in vitro) techniques were applied to assess the contribution of MITF and Mcl-1 proteins in the MFLX-induced anti-proliferative and pro-apoptotic effects on the MDA-MB-231 breast cancer cells.

**Results:**

We indicated the ability of MFLX to form complexes with MITF and Mcl-1 as well as the drug’s capacity to affect the expression of the tested proteins. We also showed that MFLX decreased the viability and proliferation of MDA-MB-231 cells and induced apoptosis via the intrinsic death pathway. Moreover, the analysis of the cell cycle progression revealed that MFLX caused a block in the S and G2/M phases.

**Conclusions:**

We demonstrated for the first time that the observed effects of MFLX on MDA-MB-231 breast cancer cells (growth inhibition and apoptosis induction) could be related to the drug’s ability to interact with MITF and Mcl-1 proteins. Furthermore, the presented results suggest that MITF and Mcl-1 proteins could be considered as the target in the therapy of breast cancer.

**Graphical abstract:**

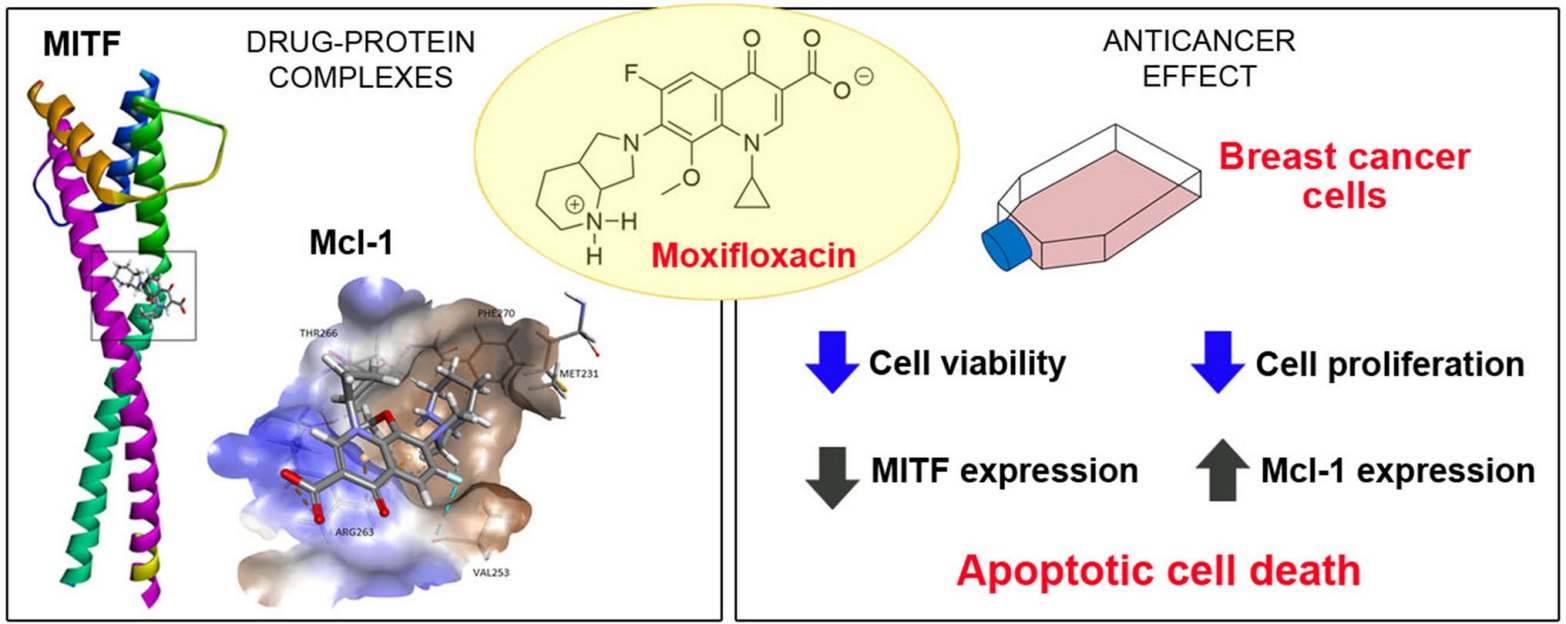

**Supplementary Information:**

The online version contains supplementary material available at 10.1007/s43440-022-00407-7.

## Introduction

Breast cancer is the most commonly diagnosed cancer and the leading cause of cancer-related death in females [1]. Triple-negative breast cancer (TNBC) lacks estrogen and progesterone receptors, and HER2 overexpression, and is characterized by the highest rates of metastatic disease and the poorest overall survival of all breast cancer subtypes [2, 3]. Currently, chemotherapy is the primary established systemic treatment for patients with TNBC in the early and advanced stages of the disease. However, the resistance to conventional anti-cancer drugs, the lack of effective targeted therapies, and the poor prognosis encourage intensive research to develop additional and better systemic treatment options for patients with TNBC [2, 4].

Currently, fluoroquinolones (FQs) remain one of the most important antibacterial agents. The mechanism of their antibacterial action is based on the inhibition of enzymes (gyrase and topoisomerase IV) that are pivotal for the replication of bacterial DNA. Moxifloxacin (MFLX) belongs to the IV generation of FQs and is widely used in the treatment of upper respiratory tract diseases as well as infections resistant to treatment with β-lactam antibiotics or macrolides [5, 6]. Recent studies indicated the potentially anti-cancer characteristics of FQs. However, scientific literature describes only several cases of the potential use of MFLX in the field of the potential cancer therapy. As a result of the modulatory effect on the cell cycle and the induction of apoptosis, MFLX had already been shown as a cytotoxic and anti-proliferative agent on MIA PaCa-2 and Panc-1 pancreatic cancer cells [7], U87MG glioblastoma cells [8], as well as on C32 and COLO829 melanoma cells [9].

Mitochondrial-dependent apoptosis is strictly regulated by members of the Bcl-2 family of proteins. While the anti-apoptotic Bcl-2 proteins, such as Mcl-1, affect apoptosis by blocking the mitochondrial release of cytochrome c, the pro-apoptotic Bcl-2 family members act by promoting such a release. The balance between the pro- and anti-apoptotic proteins determines initiation of apoptosis [10]. Scientific data have revealed the elevated level of Mcl-1 protein expression in breast cancer. Moreover, recent evidence from in vitro experiments suggests a significant role for Mcl-1 in breast cancer cell survival, particularly in triple-negative breast cancers [11]. Taking into account the above data, Mcl-1 may be considered as a promising molecular target for the treatment of TNBC.

Microphthalmia-associated transcription factor (MITF) promotes cell differentiation and proliferation and has a pro-survival function [12, 13]. MITF is frequently expressed in melanoma and has a critical role in the formation and progression of this type of cancer. During an important gene expression study of freshly resected human breast cancer specimens, the tumors expressed a group of genes (including MITF) that are expressed in melanocytic lineage cells [14]. Due to the fact that inhibition of MITF decreases cell growth in melanoma [15], this protein is worth examining as a target molecule for the TNBC therapy. Moreover, MITF promotes cell proliferation, migration, and invasion through YAP signaling [16]. In addition, YAP (Yes-associated protein) is a key effector of the Hippo pathway and an important regulator of cellular proliferation and survival [17]. Thus, YAP/Hippo signaling may promote the progression of TNBC [18], which makes MITF/YAP inhibition a target of much interest for cancer prevention and treatment.

Previously, applying both in silico and in vitro experimental models, we demonstrated that interaction between ciprofloxacin and MITF and Mcl-1 could affect viability and apoptosis of melanoma cells [19]. Encouraged by these findings and determined to explore the research project describing the new signaling pathways underlying fluoroquinolones’ anti-cancer activity, we applied molecular docking (in silico), fluorescence image cytometry, and Western blot (in vitro) panel of experiments to investigate, for the first time, the main role of MITF and Mcl-1 proteins in the MFLX-induced anti-proliferative and pro-apoptotic effects on the breast cancer cells. The human MDA-MB-231 cell line was used as an in vitro model system—it is commonly applied as a research model of triple-negative breast cancer.

## Materials and methods

### In silico analysis

The 3D structure of moxifloxacin in the lowest energy conformation was generated in Gaussian 16 (revision A.03) program [20, 21] at the density functional theory (DFT) level with the B3LYP, and 6–311 + G(d,p) basis sets. The X-ray coordinates from the Cambridge Crystallographic Data Centre (CCDC ID: ABABIQ) were used as the input 3D model.

Crystal structures of macromolecules used in molecular docking studies were obtained from the Protein Data Bank (https://www.rcsb.org/). In the analysis, we used 3D models of induced myeloid leukemia cell differentiation protein (PDB ID: 4WMV) and the microphthalmia-associated transcription factor (PDB ID: 4ATH). Missing or incomplete side chains of MITF were restored using the SWISS-MODEL server (https://swissmodel.expasy.org/) [22, 23]. Optimization of the 3D model geometry was done by the optimization algorithm in YASARA Energy Minimization Server (http://www.yasara.org/-minimizationserver.htm) [24]. AutoLigand module implicit in AutoDockTools was utilized to determine the ligand-binding site in protein [25].

Genetic Optimization for Ligand Docking (GOLD) 5.6.3 [26] was selected for the molecular docking studies. The region of the protein model applied for GOLD docking was defined as all residues of Mcl-1 within the 6 Å of the reference ligand or the coordinates fixed as X = 30.337, Y = 38.818, and *Z* = − 10.875 for MITF. Other parameters were set at default values and the complexes were submitted to 100 genetic algorithm runs using the GoldScore fitness function and CHEMPLP protocol as rescoring functions. After calculations, only the 10 highest-scored pose was returned as a docking result for ligand–cavity configuration. The obtained results were ranked by their score value and arbitrary units (a.u.) presented in GOLD. The visualization of molecular docking details was performed using the BIOVIA Discovery Studio virtual environment [27].

### In vitro studies

#### Reagents

Avelox solution for infusion (1 bottle of 250 ml containing 400 mg moxifloxacin as hydrochloride) was obtained from Bayer Healthcare Pharmaceuticals Inc. (Germany). Growth medium DMEM, penicillin G, amphotericin B, neomycin sulfate, fetal bovine serum (FBS), and trypsin/EDTA were purchased from Cytogen (Poland). Cell Proliferation Reagent WST-1 was produced by Roche GmbH (Germany). Staining reagents: DAPI (1 µg/ml), 0.1% Triton X-100 in PBS; VitaBright-48 (VB-48), propidium iodide (PI) and acridine orange (AO) solution; JC-1 (200 µg/ml in DMSO); Hoechst 33,342 (500 µg/ml), PI (500 µg/ml); NC-Slide A2 and A8; Via-1-Cassette were obtained from ChemoMetec (Denmark). Annexin V-CF488A conjugate and Annexin V binding buffer were obtained from Biotium Inc. (USA). Antibodies: anti-Mcl-1 (4572, polyclonal antibody that detects an endogenous level of human Mcl-1, source: rabbit), anti-MITF (D5G7V, monoclonal antibody that recognizes an endogenous level of total MITF protein, source: rabbit), and anti-GAPDH (14C10, rabbit monoclonal antibody) were obtained from Cell Signaling (USA). Anti-Rabbit IgG (A154), RIPA Buffer, Tween-20, and PVDF membranes were purchased from Sigma-Aldrich Inc. (USA). Pierce BCA Protein Assay Kit, penicillin G, amphotericin B, and ECL Western Blotting Substrate were purchased from Thermo Fisher Scientific (USA).

### Cell culture

The human epithelial metastatic breast cancer cell line MDA-MB-231 was obtained from the ATCC (ATCC HTB-26) and cultured in a high-glucose DMEM medium supplemented with 10% FBS, penicillin G (10 000 U/ml), amphotericin B (0.25 mg/ml), and neomycin (10 µg/ml) at 37 ˚C in 5% CO_2_. The cells from passages 6–9 were utilized to perform experiments.

### Immunoblotting

The Western blot analysis was performed according to a previously described protocol [19, 28]. GAPDH was utilized as a loading control. MDA-MB-231 cells after 12 h of incubation with MFLX at concentrations of 0.5 mM and 1.0 mM were lysed in RIPA buffer with protease and phosphatase inhibitors. The lysates were incubated on ice for 30 min. Protein concentration was quantitated spectrophotometrically (Denovix DS-11) using the BCA protein assay. As the next step, the protein lysates (45 μg/lane) were separated on SDSPAGE and then transferred onto polyvinylidene fluoride membrane. The transferred membrane was incubated with primary antibodies and a secondary antibody. Immunoreactive proteins were visualized using the G:Box Chemi-XT4 Imaging System. Densitometry measurements were made using GeneTools Software (version 4.3.5). The Western blot images were cropped for presentation in each figure, and the uncropped Western blot images were provided in the Supplementary Files (figures).

### WST-1 assay

MDA-MB-231 cells were seeded into 96-well plates (2500 cells/well) and pre-incubated in DMEM for 24 h. Then, the cells were treated with MFLX (0.001–1.0 mM) for 24, 48, or 72 h. The viability of MDA-MB-231 cells was assessed by the WST-1 colorimetric assay following a previously described method [8].

### Assessment of morphological changes: microscopic observations

MDA-MB-231 cells were seeded into T-75 flasks and pre-incubated for 24 h. Then, the cells were treated with MFLX (0.5 or 1.0 mM) for 24, 48, or 72 h. The notable morphological changes in the applied experimental model were observed using an inverted microscope Eclipse TS-100-F (Nikon, Japan) and the images were taken by means of EOS Utility software. For each condition, at least six randomly selected microscopy fields, obtained with 40 × magnification, were evaluated. Figure 3d shows the images of three independent experiments. The morphological changes in MDA-MB-231 cells induced by MFLX (e.g., cell rounding, shrinkage, and detachment) were detected by the observer with regard to the control cell cultures.

### Evaluation of intracellular thiol levels

The intracellular level of GSH (glutathione in a reduced state) was measured using the NucleoCounter NC-3000 (ChemoMetec, Denmark) image cytometer following a previously described protocol [19]. In brief, the cells were treated with MFLX (0.5 mM and 1.0 mM) for 24 h and 48 h. Then, the cells were stained with a staining solution containing VB-48, PI, and AO, and analyzed using NucleoView NC-3000 software.

### Annexin V staining

The analysis was performed according to a previously described protocol [9, 29]. In brief, following the 48 h incubation of MDA-MB-231 cells with MFLX (0.5 mM and 1.0 mM), the cells were suspended in Annexin V binding buffer (ABB) and stained with Annexin V-CF488A conjugate and Hoechst 33,342. The cell pellets were then washed with ABB, stained with PI, and analyzed using the fluorescence image cytometer.

### Detection of mitochondrial depolarization

The mitochondrial transmembrane potential was examined cytometrically using a previously described protocol [8, 29]. In brief, the cells were incubated for 24 h, 48 h, and 72 h with MFLX (0.5 mM and 1.0 mM). Then, the cell pellets were stained with JC-1.

### Cell cycle analysis and DNA fragmentation assay

Cell cycle and DNA fragmentation analyses of MDA-MB-231 cells were performed using the NucleoCounter NC-3000 fluorescence image cytometer following a previously described protocol [30]. In brief, the cells were treated with 0.5 mM and 1.0 mM MFLX for 24 h and 48 h (cell cycle analysis) or 24 h, 48 h, and 72 h (DNA fragmentation analysis). Then, the cells were fixed with 70% cold-ethanol, stained with DAPI for 5 min at 37 ˚C, and analyzed using the NucleoView NC-3000 software. The cellular fluorescence was quantified into histograms displaying the DNA content quantification. Markers in the obtained histograms were used to identify cells in different cell cycle stages: G1/G0, S phase, and G2/M phase, respectively, or to demarcate late-apoptotic cells with fragmented DNA (sub-G1 phase).

### Statistical analysis

In all in vitro experiments, mean values were calculated from at least three separate experiments performed in triplicate (*n* = 9) ± standard error (SD). The results were analyzed using GraphPad Prism 6.01 Software. One-way and two-way analyses of variance ANOVA were performed followed by Dunnett’s test and Tukey’s post hoc test, respectively. In all cases, the statistical significance was set at *p* value < 0.05.

## Results

MFLX could interact with MITF and Mcl-1 proteins—in silico experimental panel (molecular docking analysis).

MFLX, like most fluoroquinolones, can exist as three chemical compounds: cationic, anionic, and zwitterionic, depending on the pH of the aqueous solution. At the same time, literature reports indicate that MLFX exists at physiological pH mostly in the zwitterionic form [31]. Moreover, calculations performed with the ACD/Percepta software [21] show that the content of the zwitterionic state of MFLX at physiological pH is 91%. Based on these data, the zwitterionic state of the tested fluoroquinolones was used in the conducted analysis. In the in silico experimental panel, Gaussian 16 computer code was applied to establish the 3D structure of ligand (generated in their low-energy conformation), which is required for further docking studies [20].

Members of the Bcl-2 family of proteins are pivotal regulators of apoptosis. This family consists of anti-apoptotic and pro-apoptotic members. A balance between anti-apoptotic and pro-apoptotic members dictates a cell’s fate and is mediated by the BH3 domain of the BH3-only proteins inserted into a hydrophobic groove on the surface of pro-survival proteins, including Bcl-2, Bcl-xL, and Mcl-1. The BH3 domain is an amphipathic α-helix, whose hydrophobic face recognizes four hydrophobic sub-pockets, P1, P2, P3, and P4, in the BH3-binding groove on the Bcl-2 family of proteins. Therefore, small molecules that mimic BH3-only proteins and can occupy the BH3 groove may neutralize Bcl-2-like proteins, liberate pro-apoptotic Bax/Bak, or activate them directly. These artificial BH3 mimetics are thought to disable the anti-apoptotic function and thus induce tumor cell apoptosis. It was indicated that carboxylic acid derivatives (salicylic, coumaric, in-dole-2-carboxylic, and benzothiophene-2-carboxylic acids derivatives) bind with Mcl-1 protein by the occupation of hydrophobic P2 pocket and the formation of the salt bridge with Arg263 [32–34].

**Fig. 1:**
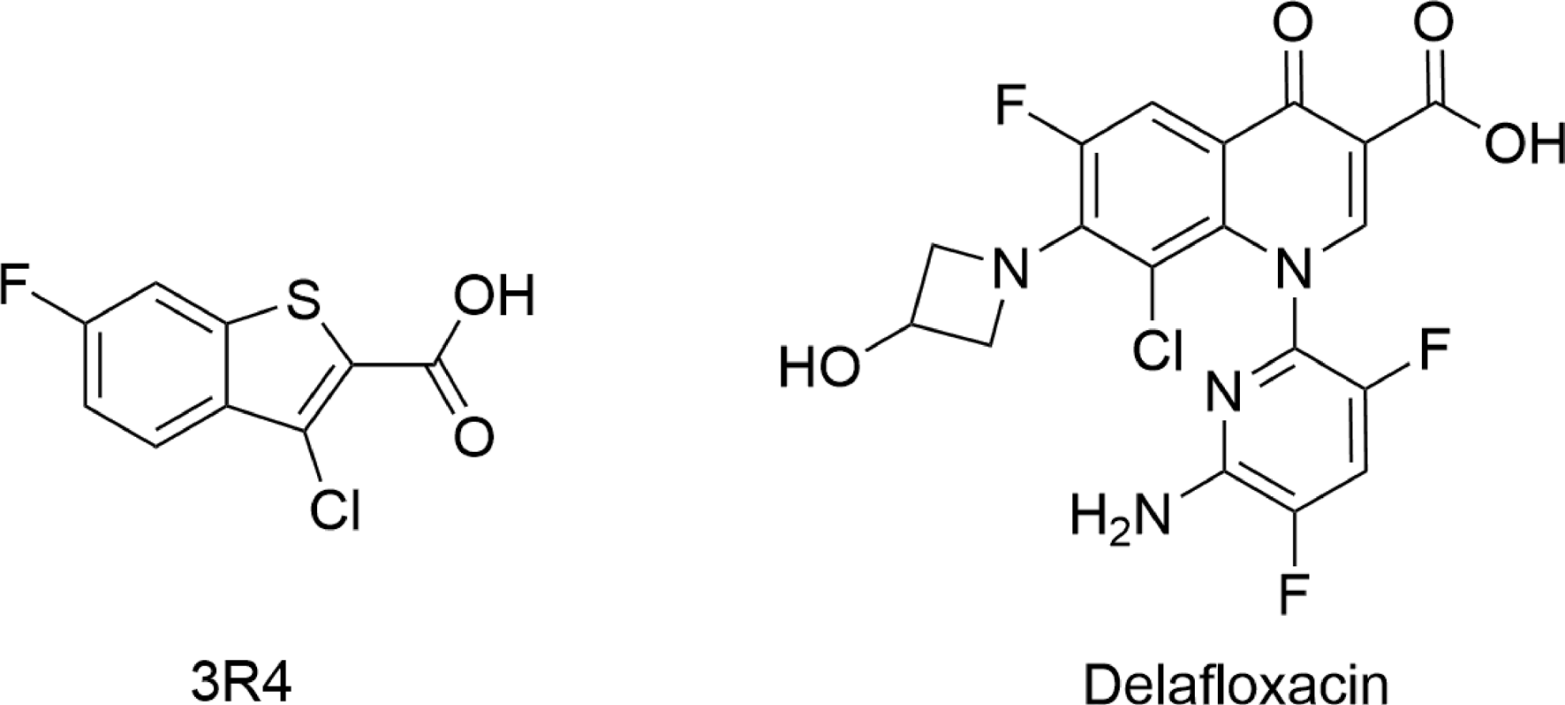
Structure of reference ligands

**Fig. 2:**
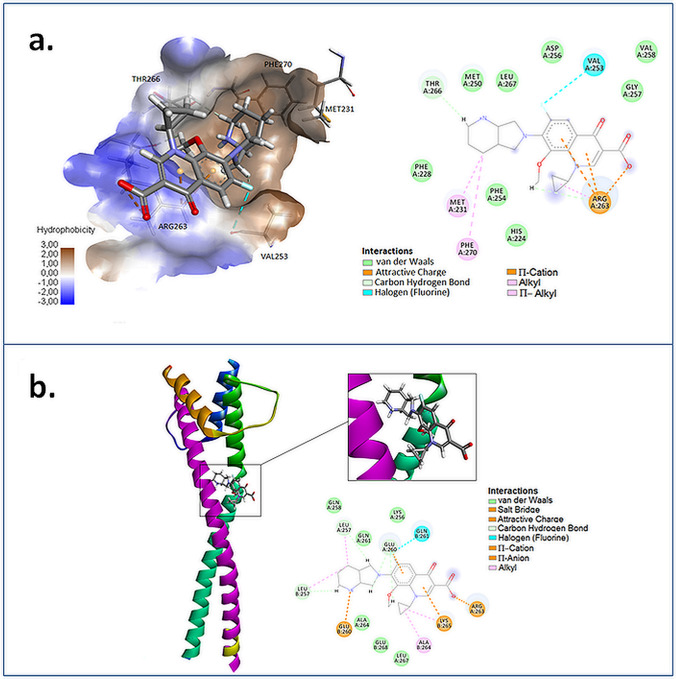
Predicted binding model of MFLX with Mcl-1 **(a)** and MITF **(b)**

We used 3-chloro-6-fluorobenzo[b]thiophene-2-carboxylic acid (3R4) (KD = 93 µM) as a model ligand in our docking study [35]. Additionally, delafloxacin, a new fluoroquinolone, was used as the reference compound (Fig. 1). To check the accuracy of the GoldScore protocol in GOLD, the cocrystallized Mcl-1 protein inhibitor was redocked into the binding site of Mcl-1 protein. MFLX and control benzothiophene-2-carboxylic acid derivative ranked by GOLD are presented in Table 1. The highest scores correspond to a strong binding affinity and the most likely ligand–protein system in cellulo. The obtained results were presented in GOLD arbitrary units (a.u.). Later on, for comparison and validation of the docking results, we used KDEEP [36] protein–ligand affinity predictor. KDEEP predicts binding affinities using state-of-the-art deep convolutional neural networks (DCNNs) and calculates the binding energy ΔG [kcal/mol] of protein–ligand complexes. In this case, the more negative the ΔG value of the binding reaction, the higher the binding affinity of the ligand for its specific target protein. The results obtained in the GOLD and KDEEP programs indicated that MFLX showed a higher GOLD fitness score and lower binding energy compared to the reference 3-chloro-6-fluorobenzo[b]-thiophene-2-carboxylic acid and delafloxacin (Table 1). A complex of MFLX with Mcl-1 revealed that the 2,8-diazabicyclo[4.3.0]nonane moiety binds deep in the p2 and p3 pockets, while the carboxylate group forms a salt bridge with Arg263 (Fig. 2a). Moreover, Arg263 forms another interaction between a positively charged nitrogen of arginine and benzene ring or 4-pyridone ring in quinolone moiety. These cation–π interactions are essentially electrostatic due to the negatively charged electron cloud of π systems. Dipolar interaction between fluorine and the amide group of Val253 is also visible, as well as a hydrophobic interaction involving an aromatic or aliphatic carbon in the receptor and an aliphatic carbon in the ligand. Subsequent weak hydrogen-bond interaction between Thr266 and hydrogen of diazabicyclononane moiety increases the stability of the ligand–receptor complex.

**Table 1:** Scoring functions and binding energy of tested complexes

Compound name	Mcl-1		MITF	
	Dock score [a.u.]	ΔG [kcal/mol]	Dock score [a.u.]	ΔG [kcal/mol
3R4	43.19	− 5.33	–	–
Delafloxacin	43.56	− 5.37	47.37	− 6.39
Moxifloxacin	44.41	− 8.26	33.91	− 4.58

MITF belongs to the basic helix–loop–helix leucine zipper transcription factors. We used the apo structure of MITF (residues 217–295). Helix α2a terminates after the first recognizable heptad repeat of the leucine zipper region–repeat 0 (residues 243–258). Next, 10-turn a helix α2b, covering the remaining heptad repeats I–V (residues 260–294), follows a marked kink at Arg259–Gln261. The second MITF protomer consists of one long helix, α2', including almost 15 turns (residues 243–294) [37]. Within the region of the kink, the established pattern of regular leucine zipper interactions is missing, inducing a hole (~ 300 Å3). In the obtained complex, the hole is filled with an MFLX molecule, as shown in Fig. 2b. The analyses of the complex, included calculations, distance measurements, and pose geometries that determined salt bridge interactions of the ligand pose with Arg263 residue (within the first protomer) and Glu260 (the second MITF protomer). Glu260 and Lys265 form another interaction between a negatively charged carboxylate group (Glu260) or positively charged nitrogen (Lys265) and benzene or 4-pyridone ring in quinolone moiety (anion–π and cation–π interactions, respectively). Moreover, numerous hydrophobic interactions, including carbon–fluorine and carbon–carbon atoms, influence the increase of the complex stability.

### In vitro experimental panel

MFLX decreases MITF protein expression and survival of MDA-MB-231 cells.

**Fig. 3:**
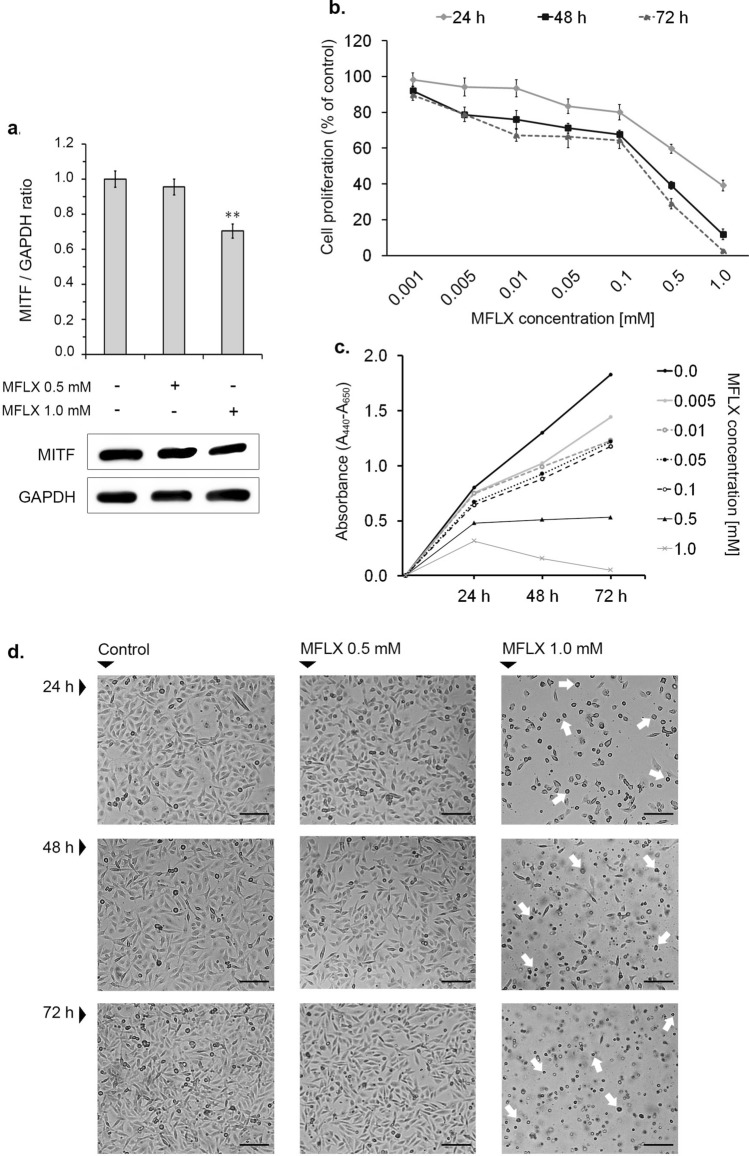
The effect of moxifloxacin on MITF protein level and MDA-MB-231 cells viability, proliferation, and morphology. Western blot analysis of basal MITF levels in MDA-MB-231 cells and MITF levels in the cells exposed to moxifloxacin for 12 h. Representative blots of three independent experiments performed in triplicate (*n* = 9) **(a)** and bar graphs, where data are presented as % of control; significance was determined using one-way ANOVA followed by Dunnett’s test; ** *p* < 0.005 The analysis of cell viability—data are expressed as % of the controls; mean values ± SD (*n* = 9). Significance was determined using two-way ANOVA followed by Tukey’s post hoc test **(b)**. The analysis of cell proliferation; mean values ± SD from three independent experiments performed in triplicate (*n* = 9) are presented **(c)**. The micrographs of MDA-MB-231 cells were taken through a light inverted microscope (scale bar 100 µm) **(d)**

To investigate whether the demonstrated in silico capacity of MFLX to form complexes with MITF protein is reflected in in vitro experimental model, the expression of MITF protein in drug-treated MDA-MB-231 cells was examined. Our results indicated a significant MITF protein expression in the tested cell line (Fig. 3a, S1, and S3). We found that the 12-h treatment of MDA-MB-231 cells with MFLX significantly decreased the expression of MITF protein, by about 30% for 1.0 mM of MFLX (a one-way ANOVA: F (2, 24) = 39; *p* < 0.001). In the next step, we verified whether the observed MITF downregulation could affect breast cancer cell viability and proliferation. As presented in Fig. 3b (a two-way ANOVA interaction: F (12, 168) = 161.2; *p* < 0.0001), MFLX in concentrations of 0.05 mM—1.0 mM resulted in a statistically significant decrease in cell viability by 17%—61% in the first 24 h, whereas after 48 and 72 h, the statistically significant decrease in cell viability (by 8% to 89% for 48 h and 11% to 98% for 72 h) was observed in all studied drug concentrations. According to the results presented in Fig. 3c, MFLX inhibited the proliferation of MDA-MB-231 cells in a time- and concentration-dependent manner. It was found that the proliferation was inhibited by the drug from a concentration of 0.5 mM. In turn, the statistically significant reduction in the cell number during the 72-h incubation was observed only in the concentration of 1 mM.

Our microscopic observation, focused at morphological changes detection in the tested experimental model, was assessed by the observer with regard to the control cell cultures (Fig. 3d). It was found that the exposure of MDA-MB-231 cells to MFLX in the concentration of 1.0 mM for 24 h, 48 h**,** and 72 h resulted in notable changes in the culture, i.e., cell rounding, shrinkage, and detachment, when compared to the controls.

### MFLX attenuates the GSH level in breast cancer cells

**Fig. 4:**
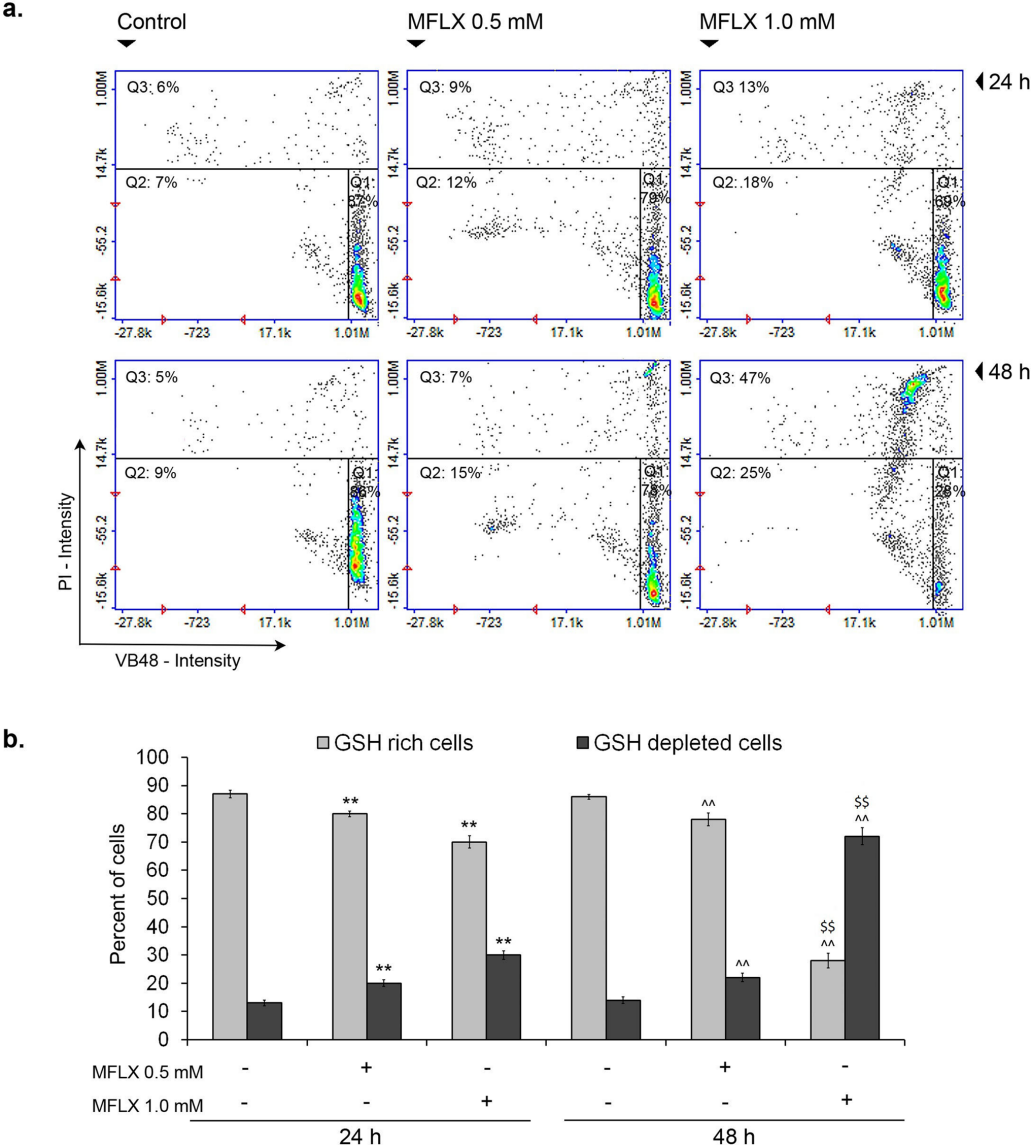
The effect of moxifloxacin on the cellular GSH level in MDA-MB-231 cells. The histograms are representative of three independent experiments (n = 9); Q1—healthy (GSH-rich) cells; Q2 + Q3—GSH-depleted cells **(a)**. Significance was determined using two-way ANOVA followed by Tukey’s post hoc test; **p < 0.005 vs 24-h control, ^^*p* < 0.005 vs 48-h control, $$ *p* < 0.005 vs 24-h corresponding sample **(b)**

As shown in Fig. 4a and b, MFLX decreased the cellular level of glutathione in its reduced state (GSH). After treatment of MDA-MB-231 breast cancer cells with the tested drug in concentrations of 0.5 mM and 1.0 mM for 24 h or 48 h, the percentages of GSH-depleted cells (PI-negative/VB-48-negative cells and PI-positive/VB-48-negative cells–Q2 and Q3) increased from about 13%, 14% (control) to approx. 21% and 31% (24 h incubation time), or 22% and 72% (48 h incubation time), respectively (a two-way ANOVA interaction: F (2, 48) = 2712; *p* < 0.0001). Simultaneously, the amount of GSH reaching cells (PI-negative/VB-positive cells–Q1) decreased from about 87% and 86% (control) to approx. 79 and 69% (24 h incubation time) or 78% and 28% (48 h incubation time), respectively (a two-way ANOVA interaction: F (2, 48) = 3668; *p* < 0.0001).

MFLX increases Mcl-1 expression and induces apoptosis and DNA fragmentation in MDA-MB-231 breast cancer cells.

**Fig. 5:**
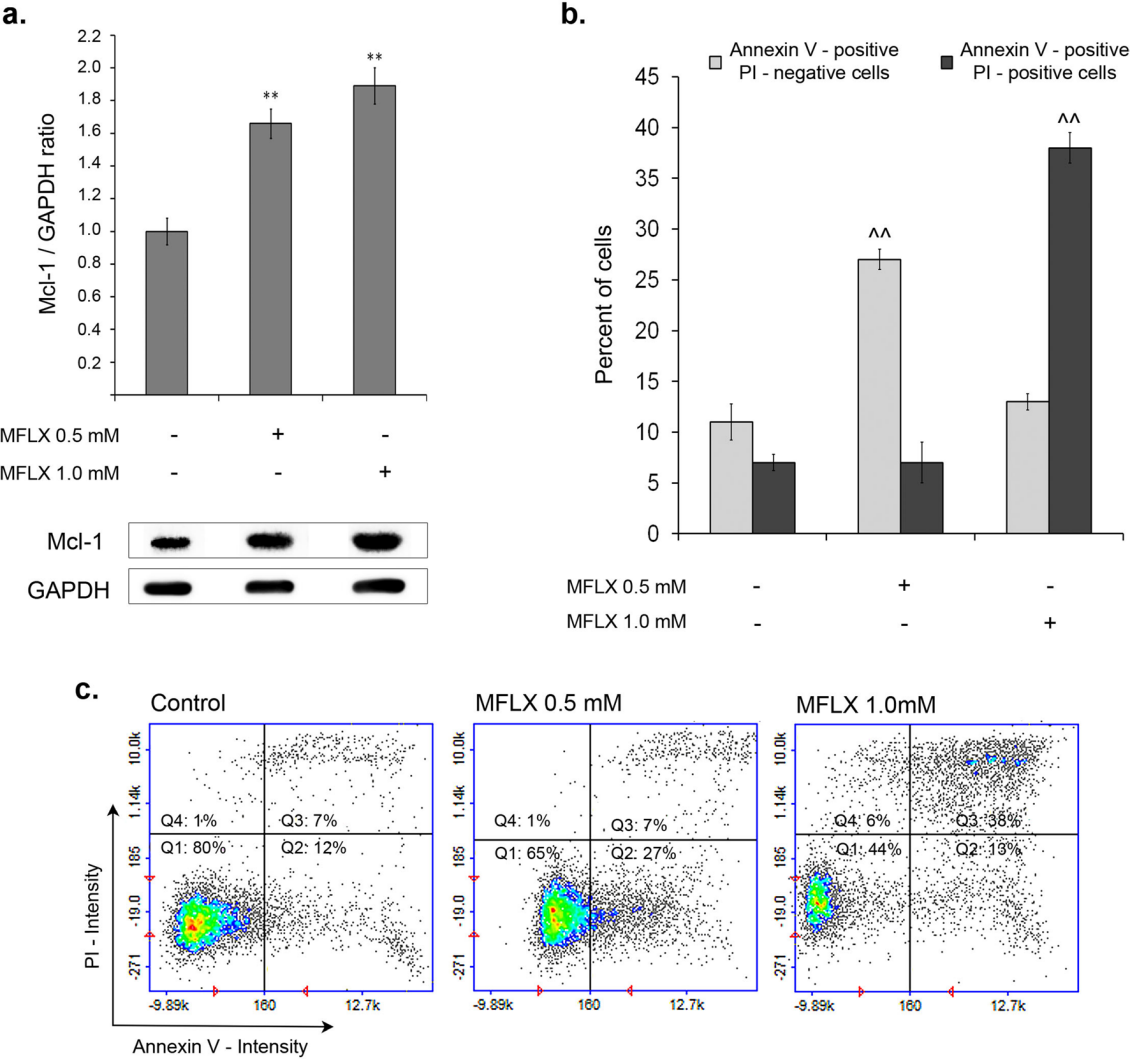
The effect of moxifloxacin on Mcl-1 protein level and apoptosis induction in MDA-MB-231 cells. Western blot analysis of basal Mcl-1 level in MDA-MB-231 cells and Mcl-1 levels in the cells exposed to moxifloxacin for 12 h. Representative blots **(a)** and bar graphs, where data are presented as % of control; significance was determined using one-way ANOVA followed by Dunnett’s test; ** *p* < 0.005. Annexin V assay; ^^*p* < 0.005 vs 48-h control **(b)**. Scatter plots demonstrating changes in Annexin V-CF488A/PI intensity of MDA-MB-231 cells—the graphs are representative of three independent experiments (*n* = 9); Q1—healthy cells; Q2—early apoptotic cells; Q3—late-apoptotic cells, and Q4—necrotic cells **(c)**

To check whether the demonstrated in silico capacity of MFLX to form complexes with Mcl-1 protein is reflected in the in vitro experimental model of human breast cancer, the expression of Mcl-1 protein in drug-treated MDA-MB-231 cells was assessed. We observed a significant Mcl-1 protein expression in the studied cell line (Fig. 5a, S2, and S3) and revealed that MFLX up-regulated Mcl-1 expression in MDA-MB-231 cells [a one-way ANOVA: F (2, 24) = 38; *p* < 0.001]. The exposure of breast cancer cells to the drug in concentrations of 0.5 mM and 1.0 mM for 12 h increased the level of Mcl-1 protein by 66% and 89%, respectively, when compared to the control.

We investigated whether apoptosis or necrosis was involved in the anti-cancer mechanism of MFLX toward triple-negative MDA-MB-231 breast cancer cells using the Annexin V-CF488A/PI staining and image cytometry. The exposure of MDA-MB-231 cells to MFLX resulted in a significant enhancement in both early apoptotic (Annexin V-positive/PI-negative, a one-way ANOVA: F (2, 24) = 172; *p* < 0.0001) and late-apoptotic (Annexin V-positive/PI-positive, a one-way ANOVA: F (2, 24) = 933; *p* < 0.0001) cells (Fig. 5b, 5c). After 48 h, only the drug in the concentration of 0.5 mM caused a significant increase in the early apoptotic cell population from 12% (control) to 27%, whereas MFLX in the concentration of 1.0 mM significantly elevated the percentages of late-apoptotic MDA-MB-231 cells from 7% (control) to 38%.

**Fig. 6:**
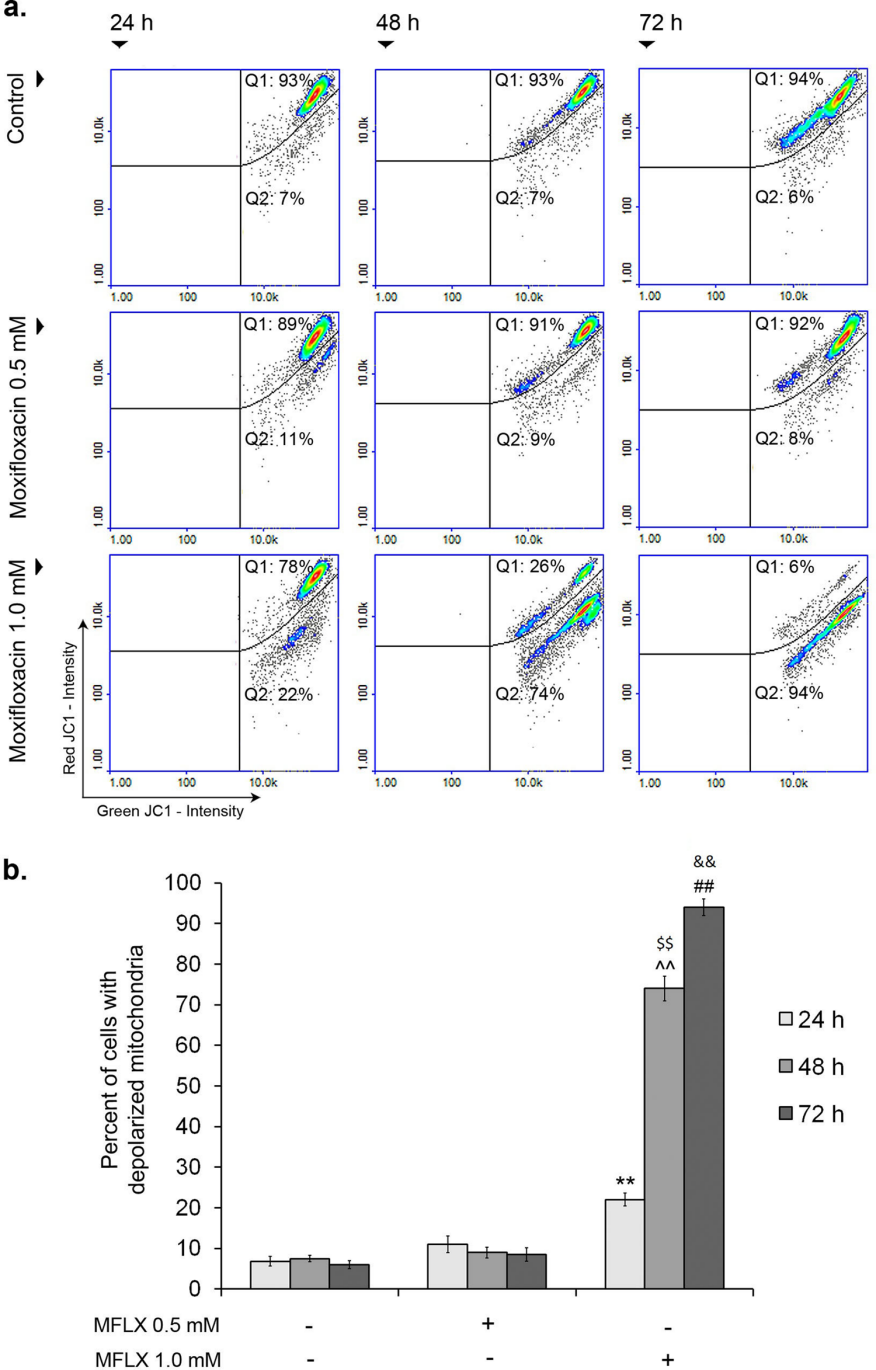
The impact of moxifloxacin on mitochondrial transmembrane potential in MDA-MB-231 cells. Scatter plots demonstrating changes in JC-1 intensity in MDA-MB-231 cells—the graphs are representative of three independent experiments (*n* = 9); Q1—cells with polarized mitochondria (healthy); Q2—cells with depolarized mitochondria (early apoptotic) **(a)**. Significance was determined using two-way ANOVA followed by Tukey’s post hoc test; **p < 0.005 vs 24-h control, ^^*p* < 0.005 vs 48-h control, ##*p* < 0.005 vs 72-h control, $$ *p* < 0.005 vs 24-h corresponding sample, && *p* < 0.005 vs 48-h corresponding sample **(b)**

In the present study, to demonstrate the mitochondrial involvement in the MFLX-induced apoptosis, a mitochondrial membrane potential (MMP) in MDA-MB-231 cells was analyzed (a two-way ANOVA interaction: F (4, 72) = 2724; *p* < 0.0001). As shown in Fig. 6a and b, a population of cells with decreased MMP after exposure to MFLX (1.0 mM for 24 h, 48 h, and 72 h) significantly increased to 22%, 74%, and 94%, respectively (the value for the controls: approx. 7%). The drug in the lower concentration had no impact on MMP in MDA-MB-231 breast cancer cells.

**Fig. 7:**
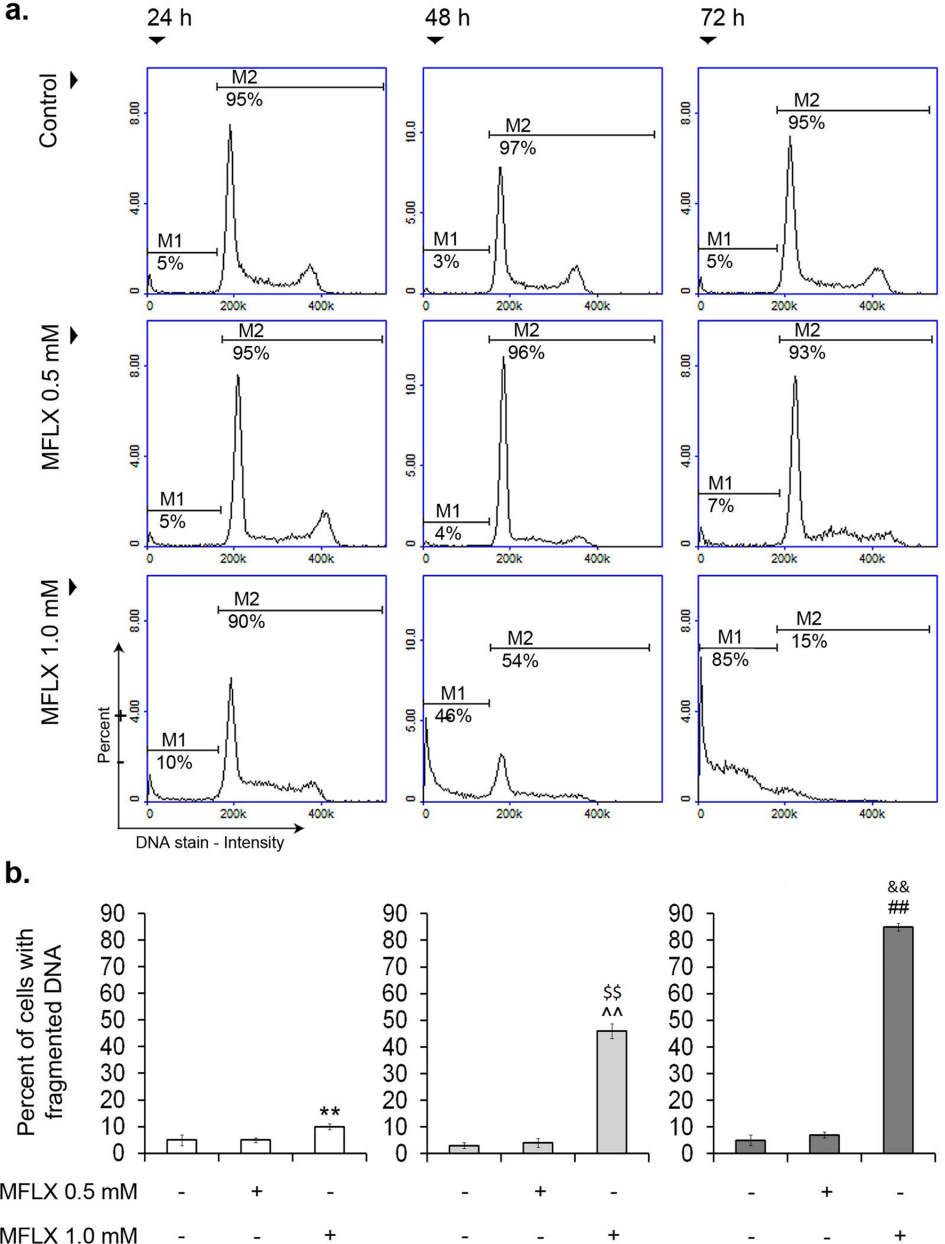
The analysis of DNA fragmentation in breast cancer cells treated with moxifloxacin. The histograms are representative of three independent experiments; M1—cells with less than 1 DNA equivalent (cells with fragmented DNA); M2–cells with 1 or more than 1 DNA equivalent **(a)**. Significance was determined using two-way ANOVA followed by Tukey’s post hoc test; (*n* = 9); **p < 0.005 vs 24-h control, ^^*p* < 0.005 vs 48-h control, ##*p* < 0.005 vs 72-h control, $$ *p* < 0.005 vs 24-h corresponding sample, && *p* < 0.005 vs 48-h corresponding sample **(b)**

DNA fragmentation is one of the key hallmarks of apoptotic cell death [38]. According to the obtained results (Fig. 7a, b), we revealed the induction of DNA fragmentation in MDA-MB-231 cells after the treatment with MFLX in concentrations of 1.0 mM for 24 h, 48 h, and 72 h (a two-way ANOVA interaction: F (4, 72) = 4759; *p* < 0.0001). The percentages of cells having less than one DNA equivalent increased from about 5% (control) to 10%, 46%, and 85%, respectively. Exposure of cells to MFLX in the concentration of 0.5 mM had no effect on DNA fragmentation in the analyzed cell line.

### MFLX disrupts the cell cycle in breast cancer cells

**Fig. 8:**
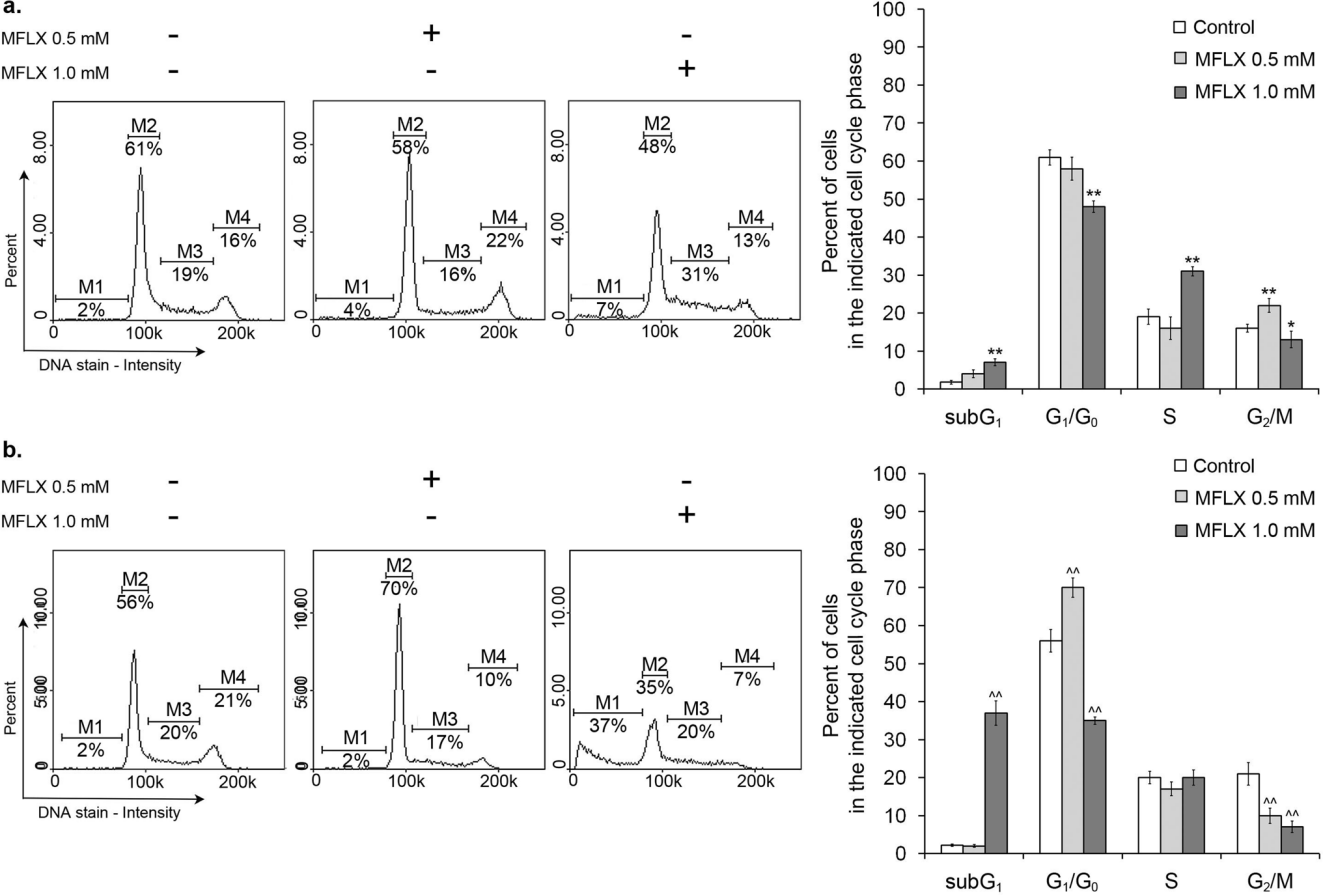
The effect of moxifloxacin on cell cycle distribution of MDA-MB-231 cells after **a** 24 h and 48 h **b** incubation time. The histograms are representative of three independent experiments (*n* = 9); M1—sub-G1 phase; M2—G1/G0 phase; M3—S phase; M4—G2/M phase. Significance was determined using two-way ANOVA followed by Tukey’s post hoc test; *p < 0.05; **p < 0.005 vs 24-h control, ^^*p* < 0.005 vs 48-h control, $$ *p* < 0.005 vs 24-h corresponding sample

The results from cytometric cell cycle analysis (Fig. 8a, b) indicated that MFLX, depending on the used drug concentration, caused both G2/M (0.5 mM) and S (1.0 mM) phase arrest in MDA-MB-231 breast cancer cells. After 24 h, the percentages of G2/M (a two-way ANOVA interaction: F (2, 48) = 465.9; *p* < 0.0001) and S (a two-way ANOVA interaction: F (2, 48) = 4357; p < 0.0001) fraction increased from 16% (control) to 22% and 19% (control) to 31%, respectively. The extension of incubation time up to 48 h resulted in a significant increase of cells in the sub-G1 phase from 2 to 37% for 1.0 mM MFLX [a two-way ANOVA interaction: F (2, 48) = 3045; *p* < 0.0001].

## Discussion

Fluoroquinolones—broad-spectrum, systemic antibacterial agents—are widely used in the treatment of variety of bacterial infections (e.g., respiratory and urinary tract infections). Recently, the anti-proliferative, pro-apoptotic, and anti-metastatic potential of these drugs has been revealed using various experimental models. Thus, repositioning fluoroquinolones into anti-cancer drugs seems to be a promising concept. A major concern regarding the use of fluoroquinolones as anti-cancer agents is that their mechanism of action and sensitivity varies against different types of cancer cells [39]. Therefore, specific research is still needed to verify the efficacy of fluoroquinolone derivatives against particular cancer types. Following the literature review, the current study is the first that determines the role of MITF and Mcl-1 proteins in the MFLX-induced cellular and molecular cascade underlying the drug’s cytotoxic and pro-apoptotic effect on triple-negative MDA-MB-231 breast cancer cells.

The in silico analysis revealed that MFLX forms complexes with MITF protein where Arg263 and Glu260 were found to be the first and the second promoters, respectively. Encouraged by the data concerning molecular docking analysis, in the next stage of the study, we examined whether moxifloxacin could interact with MITF protein in the in vitro experimental model with a subsequent impact on cell viability and proliferation. The tested drug significantly suppressed the expression of MITF at the protein level and decreased the viability and proliferation of MDA-MB-231 cells. Similarly, Satoshi et al. [40] showed that reduction of MITF protein expression resulted in growth inhibition of melanoma cells. In the current study, after exposure of breast cancer cells to MFLX for 24, 48, and 72 h, the EC50 (concentration reducing cells viability by 50%) were estimated to be 0.61 mM, 0.18 mM, and 0.12 mM, respectively, indicating that the cytotoxic effect of MFLX toward MDA-MB-231 cells is time-dependent. In our earlier study**,** we examined the effect of MFLX on normal human melanocyte viability [41]. The use of the drug in the range of concentrations from 0.001 to 0.5 mM did not affect the viability of normal cells. In our current study with the use of the TNBC in vitro model, MFLX in the concentration of 0.05 mM resulted in a statistically significant decrease in the cell viability by about 20% in the first 24 h. Therefore, it could be stated that moxifloxacin exerts higher cytotoxic activity in the case of TNBC cells. Patitungkho et al. [42] demonstrated that nitrogen adducts of the moxifloxacin–copper complexes could affect breast cancer cell lines without any toxicity toward non-tumorigenic breast epithelial cells. In addition, the analyzed fluoroquinolone antibiotics enhance (i) etoposide-induced cytotoxic, apoptotic, and anti-topoisomerase II effects in a human colon carcinoma cell line [43], (ii) anti-proliferative and apoptotic effects of etoposide in human acute monocytic leukemia THP-1 and human acute T-cell leukemia Jurkat cells [44], and (iii) camptothecin-induced cytotoxic and anti-topoisomerase I effects [45]. Furthermore, moxifloxacin was found to enhance the anti-neoplastic/anti-angiogenic activity of ineffective doses of irinotecan in HT-29 cells and colon carcinoma xenograft model [46]. Therefore, the data presented herein and obtained by other researchers allow for possible novel insight into the therapeutic properties of this drug in further in vivo studies that could strengthen the findings reported in the present study.

It is well established that reactive oxygen species play a significant role in the apoptotic process [47] and that the pro-oxidants agents may selectively target tumor cells [48, 49]. Oxidative stress or redox status shifts may cause cell transition from quiescent to a proliferative status, growth-arrested, or cell death activation according to the duration and the extent of the redox imbalance. ROS may cause single- and double-strand DNA fragmentation, may decrease mitochondrial transmembrane potential and may associate with permeability alterations, and facilitate the release of death-related molecular signals [50]. Reduced glutathione is crucial for the regulation of cellular redox homeostasis and is taken into consideration as an anti-cancer drug target [51]. Various effective anti-cancer agents were demonstrated to reduce intracellular GSH levels by oxidizing or inducing its extracellular export [52]. Moreover, elevated levels of GSH and resistance to chemotherapeutic agents were observed, e.g., to platinum-based anti-neoplastic drugs, alkylating agents, and anthracyclines. In addition, cancer cells containing reduced GSH levels were found to be much more sensitive to the effects of γ-irradiation than control cells [50]. In the present study, MFLX was found to induce GSH depletion in MDA-MB-231 breast cancer cells suggesting the role of ROS generation in the mechanism underlying the drug’s pro-apoptotic effect. In our earlier study [9], we demonstrated that the response of amelanotic (C32) and melanotic (COLO829) melanoma cells to MFLX treatment was associated with the participation of the drug-induced oxidative stress and subsequent decrease of the intracellular GSH level. The obtained results showed that preincubation of melanoma cells with vitamin c (1 µg/ml) for 24 h resulted in a reduction of the MFLX cytotoxic effect, which explains the role of oxidative stress in the mechanism underlying GSH depletion and subsequent apoptosis induction.

Evasion of apoptosis may play a significant role in the resistance of cancer cells to conventional therapeutic regimens. Nevertheless, like a double-edged sword, every defect or abnormality along the apoptotic pathways may also be an interesting target for cancer treatment [10, 11]. In the current study, the ability of MFLX for binding to Mcl-1 protein was demonstrated using the in silico molecular docking analysis with a salt bridge between the carboxylate group of the drug and Arg263 of Mcl-1 protein. Moreover, dipolar interactions between the fluorine of MFLX and the amide group at Val253 play a significant role in the Mcl-1–MFLX complex formation. Similarly, Tron et al. [53] demonstrated Arg263 of Mcl-1 as an important hot-spot for binding of AZD5991, the Mcl-1-specific inhibitor. In our study, MFLX was found to increase the level of Mcl-1 protein in MDA-MB-231 breast cancer cells and activated the apoptotic signaling pathway. Previous studies [11, 54, 55] indicated the upregulation of Mcl-1 protein in various cancer cell lines after the use of the inhibitors specific to this anti-apoptotic protein. Moreover, it was shown that the use of MIM1-high and specific Mcl-1 inhibitor increased the level of Mcl-1 protein in BRAF-mutant amelanotic C32 melanoma cells with subsequent apoptosis induction [55]. The role of increased Mcl-1 level in apoptosis induction is based on the release of Bim from Bim-Mcl-1-complex. Bim is responsible for Bax/Bak activation and thus induces apoptosis [11, 54, 55]. Therefore, apoptosis induction in triple-negative MDA-MB-231 cells after MFLX exposure may also be related to the displacement of Bim from Mcl-1.

The mitochondrial membrane permeabilization is one of the key features of the mitochondrial-intrinsic apoptotic pathway [47]. In the present study, we showed that MFLX induced significant alterations in the mitochondrial membrane potential in breast cancer cells. This effect was seen especially when MDA-MB-231 cells were exposed to the drug at a concentration of 1.0 mM for 48 h and 72 h, where the percentage of depolarized/early apoptotic cells increased by 67% and 88%, respectively, when compared to the controls. Our results suggest that MFLX activated apoptosis via the mitochondrial-mediated intrinsic apoptosis pathway.

The cell cycle analysis was performed to examine the mechanism underlying both growth inhibition and the pro-apoptotic effect of MFLX on MDA-MB-231 breast cancer cells. The 24 h incubation resulted in both G2/M and S phase arrest. Interestingly, prolongation of an incubation time up to 48 h resulted in a significant increase in the fraction of cells blocked in the sub-G1 phase, confirming both (i) the ability of MFLX to inhibit MDA-MB-231 cells proliferation as well as (ii) the activation of apoptotic cell death.

In conclusion, the obtained findings provide the first evidence that MFLX may interact with MITF and Mcl-1 proteins and, consequently, induce loss of cell viability and proliferation as well as apoptosis intensification via the mitochondrial signaling pathway. In addition, our results pointed out that the inhibition of Mcl-1 and MITF activity could be considered a target in triple-negative breast cancer treatment. In this context, we believe that the present study may give further directions for the future studies concerning the synthesis of new fluoroquinolone derivatives directed to the possible ability to interact with MITF and Mcl-1 proteins. Moreover, the presented multi-directional effect of MFLX—resulting from MITF and Mcl-1 modulatory effect—could form the basis for the potential pharmacotherapy of a drug-resistant TNBC model (Graphical abstract).

## Supplementary Information

Below is the link to the electronic supplementary material.

Supplementary file1 (JPG 17 KB) Figure S1: Panel Aa, representing the data shown in Figure 3a

Supplementary file2 (JPG 18 KB) Figure S1: Panel Ab representing the data shown in Figure 3a

Supplementary file3 (JPG 18 KB) Figure S1: Panel Ac representing the data shown in Figure 3a

Supplementary file4 (JPG 16 KB) Figure S2 Panel Ba representing the data shown in Figure 5a

Supplementary file5 (JPG 16 KB) Figure S2 Bb representing the data shown in Figure 5a

Supplementary file6 (JPG 19 KB) Figure S2 Bc representing the data shown in Figure 5a

Supplementary file7 (JPG 17 KB) Figure S3: Panel ABa representing the data shown in Figure 3a, 5a.

Supplementary file8 (JPG 17 KB) Figure S3: Panel ABb, representing the data shown in Figure 3a, 5a.

Supplementary file9 (JPG 16 KB) Figure S3: Panel ABc representing the data shown in Figure 3a, 5a.

## Data Availability

The data that support the findings of this study are available from the corresponding author upon reasonable request.
