# Supplementary figures and images for: Interaction between moxifloxacin and Mcl-1 and MITF proteins: the effect on growth inhibition and apoptosis in MDA-MB-231 human triple-negative breast cancer cells
Source: Pharmacol Rep. 2022 Sep 1;74(5):1025–40. doi: 10.1007/s43440-022-00407-7 (PMC9585003; doi:10.1007/s43440-022-00407-7)

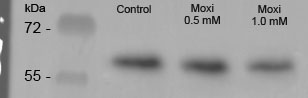

Supplement: Supplementary file 1 — Supplementary file1 (JPG 17 KB) Figure S1: Panel Aa, representing the data shown in Figure 3a [file 43440_2022_407_MOESM1_ESM.jpg]

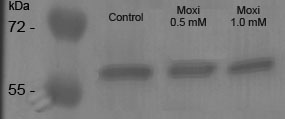

Supplement: Supplementary file 2 — Supplementary file2 (JPG 18 KB) Figure S1: Panel Ab representing the data shown in Figure 3a [file 43440_2022_407_MOESM2_ESM.jpg]

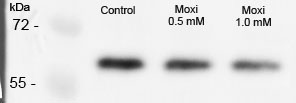

Supplement: Supplementary file 3 — Supplementary file3 (JPG 18 KB) Figure S1: Panel Ac representing the data shown in Figure 3a [file 43440_2022_407_MOESM3_ESM.jpg]

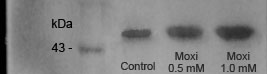

Supplement: Supplementary file 4 — Supplementary file4 (JPG 16 KB) Figure S2 Panel Ba representing the data shown in Figure 5a [file 43440_2022_407_MOESM4_ESM.jpg]

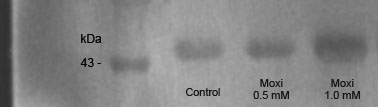

Supplement: Supplementary file 5 — Supplementary file5 (JPG 16 KB) Figure S2 Bb representing the data shown in Figure 5a [file 43440_2022_407_MOESM5_ESM.jpg]

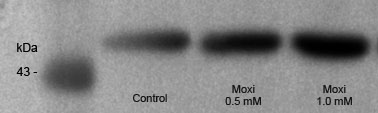

Supplement: Supplementary file 6 — Supplementary file6 (JPG 19 KB) Figure S2 Bc representing the data shown in Figure 5a [file 43440_2022_407_MOESM6_ESM.jpg]

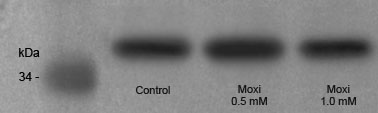

Supplement: Supplementary file 7 — Supplementary file7 (JPG 17 KB) Figure S3: Panel ABa representing the data shown in Figure 3a, 5a. [file 43440_2022_407_MOESM7_ESM.jpg]

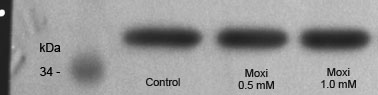

Supplement: Supplementary file 8 — Supplementary file8 (JPG 17 KB) Figure S3: Panel ABb, representing the data shown in Figure 3a, 5a. [file 43440_2022_407_MOESM8_ESM.jpg]

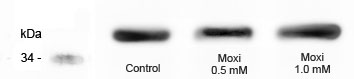

Supplement: Supplementary file 9 — Supplementary file9 (JPG 16 KB) Figure S3: Panel ABc representing the data shown in Figure 3a, 5a. [file 43440_2022_407_MOESM9_ESM.jpg]
